# A vanillic acid inducible expression system for *Trypanosoma brucei*

**DOI:** 10.1016/j.molbiopara.2016.04.001

**Published:** 2016-05

**Authors:** Jack D. Sunter

**Affiliations:** Sir William Dunn School of Pathology, University of Oxford, South Parks Road, OX1 3RE, UK

**Keywords:** TetO, *tet* operator, RNAP, RNA polymerase, TetR, tetracycline repressor, VanR, vanillic acid repressor, VanO, vanillic acid operator, *Trypanosoma brucei*, Inducible expression, Gene regulation, Vanillic acid

## Abstract

Reverse genetics in *Trypanosoma brucei* is dependent on the tetracycline inducible system for the precise control over the expression of both genes and dsRNA. Another independent inducible system for trypanosomes would enable the control of the activities of two different genes in the same cell, providing greater experimental sophistication. Here, I describe the development of the vanillic acid based inducible expression system for *T. brucei*, which operates independently of, and can be used in parallel with the tetracycline inducible system.

## Highlights

• Inducible gene expression system for *Trypanosoma brucei*.
• Addition of vanillic acid results in gene expression.
• Tetracycline and vanillic acid inducible systems are independent of each other.
• Tetracycline and vanillic acid inducible systems can be used simultaneously.

## Graphical abstract

**Figure:**
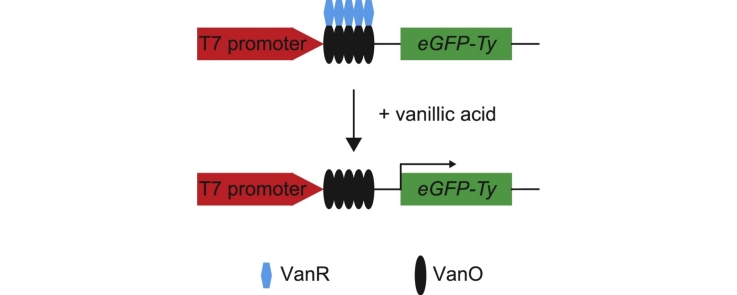

*Trypanosoma brucei* has a well annotated genome and well developed reverse genetic tools, making it a highly tractable experimental organism used for the study of a large range of biological processes such as antigenic variation and the eukaryotic flagellum [1], [2]. Precise control over the expression of exogenous genes, mutants and dsRNA is critical for reverse genetics. In *T. brucei* inducible expression is controlled by the tetracycline inducible system in the tetON configuration (Fig. 1A), where tet operator (TetO) sites are located downstream of a strong promoter and the binding of tet repressor (TetR) to the TetO sites inhibits transcription from the promoter [3], [4]. Addition of tetracycline releases this inhibition by binding to the TetR, which is now unable to bind to the TetO sites, allowing transcription to occur. The successful operation of the tetracycline inducible system requires the expression of TetR; there are now several *T. brucei* cell lines expressing these proteins and a few examples are referenced here [4], [5], [6].

Over the last 20 years the tetracycline inducible system in *T. brucei* has been extremely effective in enabling functional genomic research. Another inducible system that operated simultaneously with the tetracycline based system would greatly increase experimental flexibility, allowing the independent control of two different gene activities in the same cell, hence creating higher-order control networks. An IPTG inducible system has previously been described in trypanosomes [7]; however this was only used for the expression of a tRNA. Here, I describe the development of an inducible expression system for expression of both protein and dsRNA in procyclic form (PCF) *T. brucei* using the components that control vanillic acid metabolism in *Caulobacter crescentus*, which has successfully been used for inducible gene expression in mammalian cells [8].

Inducible gene expression using the vanillic acid system requires the expression of the vanillic acid repressor (VanR) [8]. To achieve this I modified the pSMOX plasmid, which encodes both TetR and T7 RNAP, by inserting a trypanosome codon-optimised VanR gene with a C-terminal nuclear localisation signal to create pJ1173 (Fig. 1B) [6], [9]. This single plasmid now encodes the T7 RNAP, TetR and VanR proteins and was integrated into the tubulin locus in the TREU927 PCF cell line [6]. The other essential component of the vanillic acid system is a strong promoter with vanillic acid operator (VanO) sites downstream of it (Fig. 1C). I used the pDEX877 plasmid, a derivative of the pDEX777 that encodes the blasticidin resistance gene as the starting point to create the vanillic acid inducible plasmid, pJ1271 [6]. The tetO site in pDEX877 was replaced with five VanO sites and as the VanO site contains a BamHI site, the BamHI site that is located at the 3′ end of the eGFP-Ty construct was replaced with a BglII site ensuring that the plasmid remains compatible with other plasmid systems (Fig. 1A) [5], [6], [10]. The plasmid, pJ1271 targets the 177 bp repeats like other pDEX plasmid derivatives.

pJ1271 was integrated into the 927 pJ1173 cells to give a cell line with the expression of eGFP under vanillic acid control. To determine whether this cell line (927 pJ1173 pJ1271) was able to express eGFP a range of vanillic acid concentrations were added to the cells before analysing the eGFP expression level by flow cytometry (Fig. 1D). Without vanillic acid the fluorescent signal from the 927 pJ1173 pJ1271 cells was indistinguishable from the 927 pJ1173 cell line; no protein expression was therefore detectable under non-induced conditions, demonstrating that the vanillic acid system was able to repress expression. Vanillic acid addition led to the inducible expression of eGFP with increasing vanillic acid giving higher levels of eGFP expression up to ∼125 μM vanillic acid after which eGFP expression plateaued. Based on this titration 250 μM vanillic acid was used for all subsequent inductions to ensure maximal expression.

To compare the relative expression levels between the vanillic acid and tetracycline inducible systems the pDEX877 plasmid was integrated into the 927 pJ1173 cell line to create 927 pJ1173 pDEX877. After 24 h of vanillic acid induction the 927 pJ1173 pJ1271 cell line had ∼18 fold increase in the fluorescent signal over the uninduced cell line; however, after 24 h of doxycycline induction the 927 pJ1173 pDEX877 cell line had ∼250 fold increase in the fluorescent signal over the uninduced cell line (Fig. 1E, F). The tetracycline inducible system has a greater fold increase in expression level than the equivalent vanillic acid system and this difference was seen throughout the development of the vanillic acid inducible system (data not shown); however, the 18-fold increase that the vanillic acid system can achieve should be sufficient for most experiments. A major difference between pJ1271 and pDEX877 is the 5 VanO sites next to the T7 promoter as compared to a single TetO site, and these inverted repeats may affect transcription and/or translation, resulting in lower expression levels. Furthermore, these experiments were performed with single cell lines containing either pJ1271 or pDEX877 so the difference in expression level could be due to the cell lines examined and variability may exist between different inducible cell lines. To determine whether the vanillic acid and tetracycline inducible systems were independent of each other, vanillic acid was added to the 927 pJ1173 pDEX877 cells and doxycycline was added to the 927 pJ1173 pJ1271 (Fig. 1E, F). In both cases attempted induction with the wrong chemical inducer did not result in any detectable eGFP expression. The growth rate of the cells was also unaffected by addition of doxycycline, vanillic acid or both over a 96 h period (Fig. 1G).

To show that the two induction systems could operate simultaneously, a modified version of pDEX777 called pJ1225 with eGFP replaced with dTomFP was integrated into the 927 pJ1173 pJ1271 cell line. This cell line was then treated with no chemical inducer, vanillic acid only, doxycycline only or both vanillic acid and doxycycline for 24 h; the cells were then fixed and imaged (Fig. 2A). When either vanillic acid or doxycycline was present the cells only expressed eGFP or dTomFP respectively, and when both were present the cells were able to express both eGFP and dTomFP. This demonstrates that the cells are able to support expression from both the vanillic acid and tetracycline inducible systems simultaneously and that the two systems are independent of each other. To show that the vanillic acid inducible system was capable of causing protein depletion by RNAi, a hairpin RNAi construct designed against PFR2 (Tb927.8.4970) was cloned into plasmid pJ1271 to give pJ1285, which was then integrated into the 927 pJ1173 cell line. PFR2 RNAi was induced by the addition of vanillic acid and depletion of PFR2 was followed by western blotting (Fig. 2B).

Tetracycline, IPTG and vanillic acid inducible systems have now all been described for use in PCFs [4], [7]. The tetracycline and vanillic acid systems support inducible expression of both protein and dsRNA for RNAi knockdown; however, the IPTG inducible system was only shown to support expression of tRNA [3], [7], [11]. The tetracycline inducible system will remain the mainstay of reverse genetics in trypanosome but the development of another independent inducible system opens the way to more sophisticated screens and experiments. For example the combination of the tetracycline and vanillic acid inducible system could be used to design a synthetic lethality screen to identify interacting partners of certain proteins. A cell line could be created with the genome-wide RNAi library under the control of TetR with a gene specific RNAi under the control of VanR [12]. RNAi against the specific gene of interest will be induced with vanillic acid; this will be followed by induction of the genome-wide RNAi with doxycycline. Potential interacting proteins will be identified by specific loss of those cells from the doubly induced population in comparison to the population where only the genome-wide RNAi was induced. All the plasmids developed here are freely available from the author.

## Figures and Tables

**Fig. 1:**
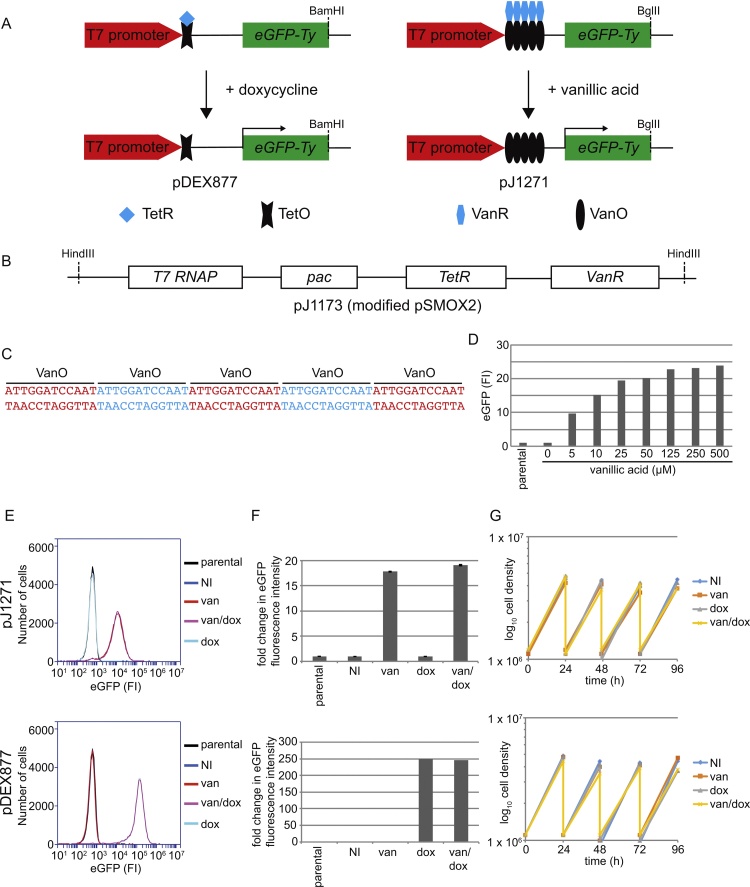
Vanillic acid inducible system is functional and independent of the tetracycline inducible system. A) Cartoon showing how the tetON and vanON inducible systems operate in *T. brucei* with the plasmids pDEX877 and pJ1271. B) Map of the modified pSMOX2 plasmid, pJ1173 that encodes the vanillic acid repressor protein (VanR), tetracycline repressor protein (TetR), T7 RNA polymerase (RNAP) and puromycin N-acetyl-transferase (pac). C) The sequence of the 5 VanO sites introduced into pJ1271. D) Titration of vanillic acid concentration shows that eGFP expression was induced after addition of vanillic acid and was tunable. Cells were incubated for 24 h with a range of different vanillic acid concentrations, and eGFP expression was measured by flow cytometry with 50,000 events captured per concentration. Vanillic acid was purchased from Sigma-Aldrich and dissolved in DMSO. E) and F) Comparison of eGFP expression induction by vanillic acid and doxycycline (final concentration of 1 μg/ml was used for all experiments) from pJ1271 and pDEX877. eGFP expression was induced by incubating the cells for 24 h with vanillic acid, doxycycline or both, and was measured by flow cytometry with 50000 events captured per induction. A histogram (E) and graph of normalised eGFP fluorescence intensity (FI) (F) is shown. This experiment was repeated three times for pJ1271, error bars are ±S.D. G) Growth curves for the 927 pJ1173 pJ1271 and 927 pJ1173 pDEX877 cell lines induced with vanillic acid, doxycycline or both over a 96 h period. Every 24 h the cells were split to 1 × 10^6^ cells/ml and fresh vanillic acid and doxycycline added.

**Fig. 2:**
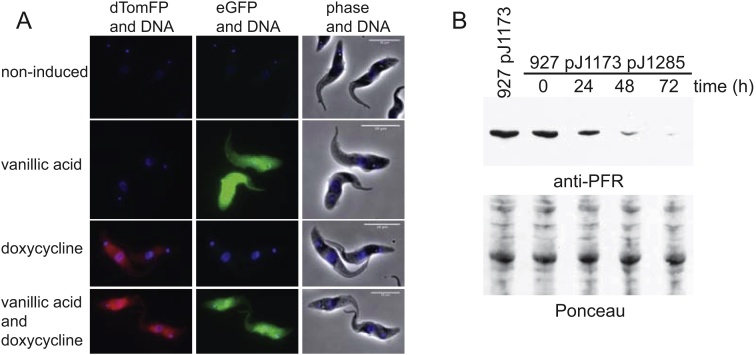
Vanillic acid and doxycycline inducible systems can be used simultaneously and vanillic acid inducible system supports RNAi knockdown. A) Images of 927 pJ1173 pJ1271 pJ1225 cells induced for 24 h with no chemical inducer, vanillic acid only, doxycycline only or both vanillic acid and doxycycline. Scale bar is 10 μm. B) Western blot of PFR2 depletion after induction of PFR2 RNAi with vanillic acid using the L8C4 antibody. 2 × 106 cell equivalents were loaded per lane with the Ponceau staining as a loading control.
